# Investigation of DNA Sequence in the Basal Core Promoter, Precore, and Core Regions of Hepatitis B Virus from Tunisia Shows a Shift in Genotype Prevalence

**DOI:** 10.5812/hepatmon.6191

**Published:** 2012-11-30

**Authors:** Rym Ayari, Yousr Lakhoua-Gorgi, Lamjed Bouslama, Imen Safar, Fatma Houissa Kchouk, Houda Aouadi, Saloua Jendoubi-Ayed, Taoufik Najjar, Kaled Ayed, Taieb Ben Abdallah

**Affiliations:** 1 Laboratory of Immunology Research Center, Kidney Transplantation and Immunopathology, Charles Nicolle Hospital, University of Tunis El Manar, Tunis, Tunisia; 2 Borj Cedria Center for Biotechnology, Hammam Lif, Tunis, Tunisia; 3 Department Gastro-Enterology, Charles Nicolle Hospital, Tunis, Tunisia

**Keywords:** Hepatitis B virus, Promoter Regions, Genetic, Mutations, Genotypes

## Abstract

**Background:**

In this study, we evaluated the prevalence of the most common mutations occurring in Enhancer II (EnhII), Basal Core Promoter (BCP), Precore (PC), and Core (C) regions of hepatitis B virus (HBV) genome.

**Objectives:**

We also investigated the correlation between HBV variants, their genotypes, and patients’ HBe antigen (HBeAg: soluble shape of the capsid antigen) status.

**Patients and Methods:**

We retrieved viral DNA from 40 serum samples of Tunisian patients positive for hepatitis B surface antigen (HBsAg) and HBV DNA, amplified the above mentioned regions using specific primers, and sequenced the corresponding PCR (polymerase chain reaction) products. For further analysis purpose, the patients were divided into two groups: Group1 including 34 HBeAg-negative patients and Group2 with 6 HBeAg-positive patients.

**Results:**

Twenty-one patients (52.5%) showed PC G1896A mutation and 11 (27.5%) carried A1762T/G1764A double mutations. These mutations were more frequent in HBeAg-negative patients than that in HBeAg-positive ones. Indeed, 58.8% of patients bearing G1896A mutation were HBeAg-negative while 16.7% were positive. In patients bearing T1762/A1764 double mutation, 29.4% were positive and 16.7% were negative. In addition, the A1896 mutation was restricted to HBV isolates that had wild-type T1858, while C1858 was rather linked to the occurrence of T1762/A1764 mutation. Interestingly, this study revealed a high frequency of genotype E. This frequency was important as compared to that of genotype D known to be predominant in the country as delineated in previous studies.

**Conclusions:**

Previous results supported and showed that HBV strains present in Tunisia belonging to genotype D and, to a lesser extent, to genotype E, were prone to mutations in BCP/ PC regions. This observation was more obvious in HBV isolates from asymptomatic chronic carriers (AsC). The high mutational rates observed in our study might result from a mechanism of viral escape that plays an important role in the loss of HBeAg.

## 1. Background

Hepatitis B infection displays an inflammatory effect that severely damages the liver. Chronic hepatitis B virus (HBV) occurs in 20% of infected subjects and leads to serious liver disease. It is estimated that more than 350 million individuals are chronically infected worldwide and many of them develop progressive diseases including liver cirrhosis (LC) and hepatocellular carcinoma (HCC) (1-3). HBV genotype, basal core promoter (BCP) and precore (PC) mutations are also associated with the development of these complications. The hepatitis B virus is characterized by its genetic and antigenic variabilities. To date, eight major genotypes (A to H) have been characterized. This genotyping was based upon a divergence over the entire nucleotide sequence of greater than 8% and most of the genotypes had distinct geographic and ethnic distributions (1, 4). It has been reported that clinical manifestations of the HBV might vary with different genotypes (5). Genotype C was associated with more severe liver disease while genotype B was associated with the development of HCC (5). Some studies reported that genotype A was found more frequently in chronically infected patients compared to genotype D which was more prevalent in patients with acute hepatitis (6). On the opposite, in India, genotype D was associated with more severe liver disease compared to genotype A (7). However, Gandhe et al. did not find the influence of genotype D on the outcome of chronic HBV infection in Indian patients. Therefore, HBV isolates, even of the same genotype, could differ in terms of virologic and clinical characteristics (8). PC region encodes HBeAg, which is an indicator for active viral replication and plays an immune regulatory role during natural infection. Enhancer II (EnhII), located in the overlapping X gene and the BCP, plays also an important role in the virus life cycle by regulating formation of 3.5 kb pregenomic RNA translating into viral core, polymerase proteins, and HBeAg (9, 10). Several epidemiological studies have shown that HBV genotype D was predominant in the Tunisian population, and frequencies of double mutation A1762T /G1764A in BCP region and common mutation G1896A in PC region of viral B genome were higher among asymptomatic chronic carriers (8, 11-13). The A1896 mutation was most commonly found in association with certain HBV genotypes (mainly D, E, and B) and was rather rare in patients infected with other genotypes. Others mutants in BCP region have also been identified independently from the genotypes in both HBeAg-negative and -positive patients.

## 2. Objectives

In the present study, our overall aim was to evaluate the prevalence of BCP/PC mutants in chronically HBV infected Tunisian patients with different clinical manifestations, and to study the correlation between HBV variants and their genotypes by taking into account their HBeAg status.

## 3. Patients and Methods

### 3.1. Patients

This retrospective study involved 40 HBsAg and HBV DNA positive Tunisian patients, composed of 26 males and 14 females with a mean age of 34.13 ± 11.15 years (range: 12 to 64 years). HBsAg and HBV DNA were detected qualitatively. The patients included 23 asymptomatic chronic carriers (AsC) of hepatitis B surface antigen (HBsAg), two patients with acute hepatitis B (AH) as confirmed by the presence of anti-HBc IgM, 13 patients with chronic hepatitis (CH) and two patients with liver cirrhosis (LC); the diagnosis was based on clinical, biological and histological criteria. All subjects were unrelated and were treated at the Department of Gastroenterology of Charles Nicolle hospital in Tunisia from 2004 to 2006. Data obtained from each patient included age at diagnosis, gender, ALT levels and clinical status of HBV infection. Determination of the ALT levels was based on biochemical analysis. The risk factors (transfusions, sexual transmission) involved in HBV infection and their durations were not included in this study. All patients were negative for anti-HIV (human immunodeficiency virus), anti-HCV (hepatitis C virus) and anti-HDV (hepatitis D virus) antibodies (Ab). None of the investigated patients received treatment for HBV infection before entering the study. All patients gave informed consent to participate in this study, which was approved by the Ethics Committee of Charles Nicolle Hospital in Tunisia.

### 3.2. Serologic Markers

Serum samples were tested by ELISA using commercial kits (Dia Sorin, Italy) to detect serological markers HBsAg/Ab, HBeAg/Ab and anti-HBc IgM/IgG, according to the manufacture’s protocol.

### 3.3. HBV-DNA Extraction

The HBV-DNA was extracted from serum by proteinase K and sodium dodecyl sulphate (SDS) treatment followed by phenol / chloroform / isoamylic alcohol (25/24/1) reagent and ethanol precipitation as previously described (14, 15).

### 3.4. Detection of HBV-DNA by PCR for Screening

HBV-DNA was detected by PCR DIG-Labeling mix (Boehinger Mannheim GmbH, Inc, Detroit, MI, USA) amplifying viral B genome in the C region by specific primers P1 (sense: 5’-ggAgTgTggATTCgCACT-3’, position: nt 2267-2284) and P2 (antisense: 5’-TgAgATCTTCTgggACgC-3’, position: nt 2436-2419).

### 3.5. Amplification of Precore and Core Promoter Regions by PCR

PCR was used to amplify the fragment from nucleotide 1653 to 1959, including Enh II (nt 1685-1773), BCP (nt 1742-1849) and PC regions (nt 1814-1900) by primers P68 (sense: 5’-CATAAgAggACTCTTggACT-3’, positions: nt 1653-1672) and P67 (antisense: 5’-ggCAAAAACgAgAgTAACTCCAC-3’, positions: nt 1959-1937). PCR was performed on 10 µL of extracted DNA in a 50 µL final volume of reaction mix containing 5 µL of PCR buffer 10 X, 1.5 mM MgCl_2_, 200 µM dNTP, 10 pmol of each primer and 1 U of Taq polymerase (Promega, USA). The PCR program was as follow: denaturation at 94°C for 4 min 30 sec followed by 40 cycles of denaturation at 94°C for 30 sec, annealing at 53°C for 30 sec and elongation at 68°C for 35 sec, followed by elongation at 68°C for 7 min. The amplified products were carried out on 2 % agarose gel stained with ethidium bromide and visualized under UV. In the case of positive reaction with a band at 307 bp, the samples were purified and sequenced.

### 3.6. HBV-DNA Sequencing

PCR products were purified using QIAquick™ PCR purification kit (Qiagen, Germany). Direct sequencing of purified PCR products was then carried out by ABI PRISM® BigDye™ sequencing kits (Applied Biosystems, Foster City, CA) using an ABI PRISM® 310 genetic analyzer (Applied Biosystems, Foster City, CA). Each amplicon was sequenced using the sense and antisense primers. Nucleotide sequences and deduced amino acid sequences were aligned and compared using MEGA software version 4.0 (17).

### 3.7. Determination of Viral Genotypes

The HBV genotypes were determined using two different methods:

1) Genotyping based on nucleotide sequences of BCP/PC regions using the relationship between basal core promoter, precore mutations and genotypes, which has been noted and confirmed (16, 17).

2) Genotyping determined by sequence alignment: the sequences obtained for BCP/PC regions were aligned and compared with the sequences in the international database using the BLAST algorithm. Viral genotypes were determined by sequence alignment with the selected Genebank sequences and sequences showing the highest matching scores with our sequences in an NCBI BLAST search were retained.

### 3.8. Statistical Methods

Statistical comparisons between different groups of patients were performed by non-parametric tests. A P value < 0.05 was considered statistically significant. Logistic regression models were used with the presence of HBV PC/C mutations as the response variable to evaluate the relationships with the different factors such as age, gender and ALT levels (including confounders).

## 4. Results

### 4.1. Epidemiologic and Viral Characteristics

As shown in Table 1, the patients were classified into two groups according to their HBeAg status. Patients in Group 2 were statistically younger (23.5 years) than those in Group1 (35.1 years) (P < 0.05), but there was no significant difference between gender in both groups. ALT levels were statistically higher among HBeAg-positive patients (307.5 U/L) compared to HBeAg-negative patients (77.79 U/L). All asymptomatic chronic carriers had normal levels of ALT (range: 5-55 U/L; mean: 46.89 U/L) and in patients with chronic carrier status, the cytolysis ranged from 0.5 to 7 times the normal levels of ALT with mean levels at 169.83 U/L.

**Table 1:** Clinical Characteristics of Patients

	Total (n = 40)	Group 1: HBeAg-negative (n = 34)	Group 2: HBeAg-positive (n = 6)
**Age, y, Mean ± SD**	34.13 ± 11.15	35.1 ± 10.55	23.5 ± 12.72
**Sex, Male/Female**	26 / 14	22 / 12	4 / 2
**Clinical Status **			
**AsC, No. (%)**	23 (57.5)	23 (67.5)	-
**AH, No. (%)**	2 (5)	-	2 (33.5)
**CH, No. (%)**	13 (32.5)	9 (26.5)	4 (66.5)
**LC, No. (%)**	2 (5)	2 (6)	-
**ALT, U/L, Mean **			
**Normal level: 5-55U/L**	81.86	77.79	307.5

Abbreviations: AH, Acute Hepatitis; ALT, Alanine Aminotransferase; AsC, Asymptomatic Chronic Carriers of HBsAg; CH, Chronic Hepatitis; LC, liver cirrhosis.

### 4.2. Precore and Core Promoter Region Sequences Analysis

Single nucleotide polymorphisms (SNPs) with frequencies greater than 5% were observed in different positions as shown in Table 2. In EnhII region, the frequencies of mutations a at nt 1701, G at nt 1702 and C or T at nt 1703 were 7.5%, 12.5% and 55%, respectively. At nt 1719, only 1 asymptomatic chronic carrier patient showed wild-type strain. The samples of 39 patients containing wild-type strain at position 1721, had G1719T, C1726A, T1727A and G1730C mutations. The wild-type strain for nt 1703 present in 18 samples, had the wild-type at the positions 1701, 1702, 1721, 1728 and 1740, but had mutant-type at nt 1719 (G to T), nt 1726 (C to A), nt 1727 (T to A) and nt 1730 (G to C). In EnhII/BCP regions the most frequent mutation was observed at nt 1757 (65%) followed by mutations at positions 1773 (62.5%) and 1753 (32.5%). Twenty-one out of 40 isolated strains (52.5%) showed wild-type sequence A1762/G1764 and 11 patients (27.5%) had the classic double mutation T1762/A1764 pattern of which 10 cases were in Group 1 (29.4%) with either asymptomatic chronic carriers status or chronic hepatitis disease (seven and three cases, respectively) and one case in Group 2 (16.7%) with acute hepatitis (P = 0.46). However, after adjustment for age and gender as confounders, logistic regression model did not reveal. The remaining serum samples had a mixture of mutant and wild-type sequences including A1762/A1764 in two patients with asymptomatic carrier status; T1762/G1764 in one patient with asymptomatic carrier status and one patient with chronic hepatitis; and A1762/T1764 in two asymptomatic chronic carriers, one chronic hepatitis patient and one patient with liver cirrhosis. In BCP region, all wild-type patients for position 1799 had also T at nt 1800 and GCAC wild-type strain at position 1809-1812 but were C1802/G1803 mutant variant. All observed strains who carried mutations at nt 1726 (C to A), nt 1727 (T to A or G) and nt 1730 (G to C) in EnhII region, had BCP mutation at position 1802C-1803G. In PC region, 21 out of 40 strains (52.5%) carried 1896A variant. All of them had C to T change at nt 1858. According to the status of HBeAg, a PC variant was more commonly found in Group 1 (58.8%) compared to Group 2 (16.6%). This difference was not statistically significant (P = 0.07) and multivariate analysis did not confirm this association after adjustment for age and gender. Only 20 isolates out of 34 HBeAg-negative cases were found to have G to A mutation at nt 1896. Among 6 HBeAg-positive patients, only 1 patient was found to have A1896 mutation. The frequency of this mutation was 65% (15 out of 23 patients) in HBV asymptomatic chronic carriers, 38.5% (5 out of 13 patients) in those with chronic hepatitis and in 1 of 2 liver cirrhosis patients, but was not observed in patients having acute hepatitis. Of limited number of 1858C variant virus examined in this study, none of them was found to have 1896A mutation when mutant G1896A was associated with double mutation A1850T and C1858T. At nt 1899, 28 patients (70%) showed the G wild-type sequence (22 cases in Group1 composed of 16 asymptomatic chronic carriers, four patients with chronic hepatitis and two patients with liver cirrhosis; and six cases in Group2 composed of two acute hepatitis and four chronic hepatitis patients) and 12 patients (30%), all in Group1, had the 1899A mutant common, consisting of 7 asymptomatic chronic carriers (30.4%) and five chronic hepatitis patients (55.5%). The frequency of double mutation A1896/A1899, all in Group1, was 25% (10/40 patients), consisting of six asymptomatic chronic carriers (26%) and 4 chronic hepatitis patients (44.4%). In both groups and independent from clinical status, all patients were carriers of 1850T mutant-type strain.

**Table 2:** Analysis of Different SNPs Obtained from 40 Sequenced Sera in BCP/PC Regions

Position	Variant	Patients, No. (%) (n = 40)	Gender, No.		HBeAg, No.		Status, No.			
			Male (n = 26)	Female (n = 14)	Group 1 a (n = 34)	Group 2 b (n = 6)	AsC (n = 23)	CH (n = 13)	AH (n = 2)	LC (n = 2)
**EnhII**										
**1701**	G wild-type	37 (92.5)	24	13	31	6	21	12	2	2
	A mutant common	3 (7.5)	2	1	3	0	2	1	0	0
**1702**	C wild-type	35 (87.5)	23	12	30	5	20	11	2	2
	G mutant rare	5 (12.5)	3	2	4	1	3	2	0	0
**1703**	A wild-type	18 (45)	12	6	14	4	10	6	1	1
	C mutant rare	5 (12.5)	3	2	4	1	3	2	0	0
	T mutant rare	17 (42.5)	11	6	16	1	10	5	1	1
**1719**	G wild-type	1 (2.5)	0	1	1	0	1	0	0	0
	T mutant common	39 (97.5)	26	13	33	6	22	13	2	2
**1721**	G wild-type	39 (97.5)	26	13	33	6	22	13	2	2
	A mutant common	1 (2.5)	0	1	1	0	1	0	0	0
**1722**	T wild-type	38 (95)	25	13	32	6	21	13	2	2
	A mutant	2 (5)	1	1	2	0	2	0	0	0
**1726**	A mutant common	40 (100)	26	14	34	6	23	13	2	2
**1727**	A mutant common	39 (97.5)	26	13	33	6	22	13	2	2
	G mutant rare	1 (2.5)	0	1	1	0	1	0	0	0
**1728**	G wild-type	38 (95)	24	14	32	6	21	13	2	2
	A mutant	2 (5)	2	0	2	0	2	0	0	0
**1730**	C mutant common	40 (100)	26	14	34	6	23	13	2	2
**1740**	T wild-type	38 (95)	25	13	32	6	21	13	2	2
	C mutant	2 (5)	1	1	2	0	2	0	0	0
**1741**	T wild-type	39 (97.5)	25	14	33	6	22	13	2	2
	C mutant	1 (2.5)	1	0	1	0	1	0	0	0
**EnhII/BCP**										
**1752**	A wild-type	37 (92.5)	23	14	31	6	31	13	2	1
	C mutant rare	3 (7.5)	3	0	3	0	2	0	0	1
**1753**	T wild-type	27 (67.5)	17	10	22	5	15	9	1	2
	C mutant common	13 (32.5)	9	4	12	1	8	4	1	0
**1754**	T wild-type	39 (97.5)	25	14	33	6	22	13	2	2
	G mutant rare	1 (2.5)	1	0	1	0	1	0	0	0
**1757**	G wild-type	14 (35)	11	3	13	1	10	3	1	0
	A mutant common	26 (65)	15	11	21	5	13	10	1	2
**1761**	A wild-type	36 (90)	23	13	30	6	21	12	2	1
	C mutant	4 (10)	3	1	4	0	2	1	0	1
**1762-1764**	A-G wild-type	21 (52.5)	13	8	16	5	11	8	1	1
	T-A mutant common	11 (27.5)	9	2	10	1	7	3	1	0
	A-A mutant rare	2 (5)	1	1	2	0	2	0	0	0
	T-G mutant rare	2 (5)	0	2	2	0	1	1	0	0
	A-T mutant rare	4 (10)	3	1	4	0	2	1	0	1
**1766**	C wild-type	36 (90)	23	13	30	6	21	12	2	1
	G mutant rare	3 (7.5)	2	1	3	0	1	1	0	1
**1773**	A mutant rare	1 (2.5)	1	0	1	0	1	0	0	0
	T wild-type	15 (37.5)	10	5	12	3	8	6	0	1
	C mutant common	25 (62.5)	16	9	22	3	15	7	2	1
**BCP**										
**1799**	C wild-type	38 (95)	24	14	32	6	23	12	2	1
	G mutant common	2 (5)	2	0	2	0	0	1	0	1
**1800**	T wild-type	40 (100)	26	14	34	6	23	13	2	2
**1802-1803**	CG mutant common	40 (100)	26	14	34	6	23	13	2	2
**1809-1812**	GCAC wild-type	40 (100)	26	14	34	6	23	13	2	2
**PC**										
**1817**	CAA wild-type	40 (100)	26	14	34	6	23	13	2	2
**1839**	T wild-type	37 (92.5)	24	13	31	6	21	12	2	2
	A mutant	3 (7.5)	2	1	3	0	2	1	0	0
**1842**	T wild-type	37 (92.5)	23	14	32	5	21	12	2	2
	C mutant	3 (7.5)	3	0	2	1	2	1	0	0
**1846**	T wild-type	40 (100)	26	14	34	6	23	13	2	2
**1850**	T mutant common	40 (100)	26	14	34	6	23	13	2	2
**1858**	T wild-type	37 (92.5)	23	14	32	5	21	13	1	2
	C mutant common	3 (7.5)	3	0	2	1	2	0	1	0
**1862**	G wild-type	40 (100)	26	14	34	6	23	13	2	2
**1874**	A wild-type	40 (100)	26	14	34	6	23	13	2	2
**1888**	G wild-type	40 (100)	26	14	34	6	23	13	2	2
**1896**	G wild-type	19 (47.5)	11	8	14	5	8	8	2	1
	A mutant common	21 (52.5)	15	6	20	1	15	5	0	1
**1899**	G wild-type	28 (70)	17	11	22	6	16	8	2	2
	A mutant common	12 (30)	9	3	12	0	7	5	0	0

Abbreviations: AH: Acute Hepatitis; AsC: Asymptomatic Chronic carriers of HBsAg; CH: Chronic Hepatitis; LC: Liver Cirrhosis.

^a^Group 1: HBeAg-negative.

^b^Group 2: HBeAg-positive

### 4.3. Determination of Viral Genotypes (Table 3)

**Table 3:** Determination of Viral Genotypes with Two Different Methods

	Group 1: HBeAg (-) (n = 34)			Group 2: HBeAg (+) (n = 6)		Total (n = 40)
	**AsC, No.**	**CH, No.**	**LC, No.**	**CH, No.**	**AH, No.**	
**Genotyping Method I a**						
**Genotype D **	15	4	1	1	-	21
**%**	59			16.5		52.5
**Genotype E**	6	5	1	3	1	16
**%**	35			67		40
**Genotype A**	2	-	-	-	1	3
**%**	6			16.5		7.5
**Genotyping Method II b**						
**Genotype D**	12	9	2	3	1	27
**%**	67.5			66.5		67.5
**Genotype E**	9	-	-	1	1	11
**%**	26.5			33.5		27.5
**Genotype A**	2	-	-	-	-	2
%	6			0		5

Abbreviations: AsC: Asymptomatic Chronic Carriers of HBsAg; CH: chronic hepatitis; LC: Liver cirrhosis; AH: Acute Hepatitis.

^a^Genotyping method I: Genotyping based on nucleotide sequences of BCP/PC regions.

^b^Genotyping method II: Genotyping determined by alignment with the selected GenBank sequences according to BLAST data.

1) Using the relationship between mutations and genotypes, the results of HBV genotype determination for 40 sequenced isolates were as follows:

- 21/40 (52.5%) genotype D with 1802-03CG/1858T/1896A pattern [20 cases in Group1 consisting of 15 asymptomatic chronic carriers, four patients with chronic hepatitis, and one with liver cirrhosis; and one case having chronic hepatitis in Group2]

- 16/40 (40%) genotype E with 1802-03CG/1858T/1896G sequence [12 cases in Group1 consisting of six asymptomatic chronic carriers, five patients with chronic hepatitis, and one with liver cirrhosis; and four cases in Group 2 consisting of three patients with chronic hepatitis and one with acute hepatitis]

- 3/40 (7.5%) Genotype A with 1802-03CG/1858C/1896G profile [two asymptomatic chronic carriers in Group1 and one patient with acute hepatitis in Group2].

2) The identification of HBV genotypes by BLAST for 40 analyzed sequences showed that genotype D was dominant with 67.5% (27/40) [25 cases in Group1 consisting of 17 asymptomatic chronic carriers, six patients with chronic hepatitis and two with liver cirrhosis; and two patients with chronic hepatitis in Group2], while genotype E was observed in 11 patients (27.5%) [8 patients in Group1 consisting of five asymptomatic chronic carriers and three patients with chronic hepatitis; and three patients in Group2 consisting of two patients with chronic hepatitis and one with acute hepatitis]. Only two patients (5%) showed genotype A [one asymptomatic chronic carrier in Group1 and one patient with acute hepatitis in Group2].

## 5. Discussion

The objective of this study was to investigate the prevalence and significance of BCP/PC mutations in 40 isolated strains as representative of HBV infected population in Tunisia. Samples were analyzed for the sequence corresponding to BCP (nt 1701-1812) and entire PC (nt 1817-1899) regions of hepatitis B viral genome. According to the numbering system used (10, 18-20), sequencing of PC region of our isolates showed that the DR1 segment (nt 1824-1834) and the epsilon region (nt 1850-1903) were highly conserved with only few nucleotide substitutions observed. The few mutations found in PC region were clustered at positions 1839, 1842, 1850, 1858, 1896 and 1899. These mutations are shown in Table 2. Other mutations found in the same region were mostly silent or not significant like 1817, 1846, 1862, 1874 and 1888 changes. In this study, like in our earlier report (8), we first confirmed the observations concerning common mutations scattered over the sequenced region. However, using a set of primers targeting a large region (from nt 1653 to nt 1959) of a great interest for several mutations, we observed for the first time the presence of genotype E with a high frequency. In spite of the small number of patients in Group2 compared to those in Group1, our study confirmed also that these mutations were more frequently found in HBeAg-negative patients (8). As far as common variants were concerned, similar to reports of some studies we observed that the frequency of the classic double mutation T1762/A1764 and that of the A1896 variant were more associated with HBeAg-negative than with HBeAg-positive phenotypes (8, 12, 21). These frequencies showed a striking difference between asymptomatic chronic carriers and chronic hepatitis B patients. Indeed, EnhII and BCP regions of HBV genome control the transcription of X gene, PC mRNA, and pregenomic RNA (20, 22). In addition, it was suggested that the double mutation T1762/A1764 in BCP region, down regulates HBeAg production due to the requirement of base pairing at pregenomic RNA level (20, 22, 23). Okamoto et al. speculated that the mutations in BCP region might abort transcription of PC mRNA affecting protein expression of PC gene and consequently could explain why a PC wild-type isolate might elicit an anti-HBe phenotype (24). In this study, we found that all detected strains showed mutations at nt 1726, nt 1730, nt 1802/1803, and nt 1850; the most frequent common mutation in BCP and PC regions was T1773 (97.5%), followed by C1773 (62.5%), A1896 (52.5%), T1703 (42.5%), C1753 (32.5%), A1899 (30%) and T1762/A1764 (27.5%). Among host-virus interactions which were involved in the pathogenesis of the HBV, the relevance of naturally occurring viral variants in PC/C regions was reported in some studies (25-27) and their presence was associated with the wide spectrum of clinical patterns ranging from an asymptomatic chronic state to self-limited acute hepatitis, or fulminant to chronic hepatitis with progression to liver cirrhosis and hepatocellular carcinoma (28-31). At present, it is well established that the host immune response is the key determinant influencing the course of disease and the onset of liver disease (25). Consequently, the hypothesis of an important mechanism of viral escape mutations with viral persistence was suggested (25). The fact that all our samples had a mixture of mutant and wild-type sequences but that the proportion of single nucleotide polymorphisms (SNPs) at various analyzed positions was much more important within the isolates of Group1 (HBeAg-negative patients) compared to that of Group2 (HBeAg-positive) would be in favor of this suggestion. The results of our study corroborated with those in the literature concerning mutations in BCP/PC regions of viral B genome. The A1896 variant of PC region was described in anti-HBe/HBV DNA positive patients (32). In fact, HBV strains showing this mutation cannot express the HBeAg either on the cell membrane or in the circulation (2). It was established that a change from G to A at position 1896 increases the stability of stem-loop structure of the pre-genome encapsidation sequence, if a T is present at the opposite nt 1858 position (17). The sequence analysis demonstrated that HBV with 1858T was dominant genotype among our isolates because it might stabilize the stem structure of epsilon encapsidation signal by T-A pairing. In the one hand and according to this hypothesis in PC region, our results revealed that this mutation was restricted to HBV isolates with wild-type T1858 associated with mutant common T1850 pattern. On the other hand, due to the requirement of base pairing at pregenomic RNA level, the presence of A1896 mutation was thought to be restricted to genotypes that had a T1858, as in genotypes B, C, D and E (22). This explains the high prevalence of A1896 in Asia and the Mediterranean region where the predominant HBV genotypes are B, C and D (1, 8, 17, 30), and its low prevalence in North America and Europe (27). According to these findings and using two different methods for HBV genotyping, either the relationship between mutations and genotypes noted and confirmed by Kramvis (16, 17) or sequence alignment in the international database using the BLAST algorithm, the results of 40 sequenced isolates were as previously reported for genotype D as 52.5% and 67.5% and for genotype E as 40% and 27.5%, while for genotype A it was 7.5% and 5%, respectively. The predominance of genotype D in the Mediterranean area, southern Europe and the Middle East has been reported in many studies (8, 12, 21). The low diversity of HBV genotypes noted in our study suggests that genotype D has a short evolutionary in this area. Indeed, Magnius et al. suggested that genotype D might have replaced genotype A in the Mediterranean area including North Africa. It has been found to be a more predominant genotype in the south and oriental regions than in southeastern Europe where genotype A is commonly found (33, 34). Therefore, our study revealed a shift in the genotype prevalence in Tunisia, and to our best knowledge, this was the first time that a high frequency of genotype E was observed in this part of the world. We speculate that the frequency of this genotype is potentially higher compared to genotype D which was always predominant in our country, as previously reported in many published studies (8, 11-13). The slight discordance in the results of HBV genotyping between the two used methods could be due either to the fact that genotypes E and D are genetically similar, or to the approach we used for genotyping. In fact, genotype E did not seem to be separate from genotype D in the X and C open reading frames (ORFs) (35). Our results argued in favor of this later hypothesis. Several molecular methods have been used for HBV genotyping like nucleotides sequencing in pre-S/S regions, but full length genome sequencing remains the gold standard. Although there are exceptions, as in any genotyping method, HBV strains can be accurately genotyped by sequencing two regions of HBV genome, namely, the S region and BCP/PC regions (16). During years and in the course of chronic hepatitis B evolution, the infectious stocks can mutate and, as a result, change of genotype occurs. The strong prevalence of genotype E can also be in favor of the emergence of this genotype among stocks infecting Tunisian patients. In many countries where well-known waves of migration have occurred over time, the prevalence of genotype D reflects the origin of immigrants and other patterns of migration. In conclusion, our study supported previous results that Tunisian HBV strains, belonging to genotype D and to a lesser extent to genotype E, are prone to common and minority mutations in BCP and PC regions. These variants occurred more in HBV isolates from patients with asymptomatic carrier status compared to those from chronic hepatitis patients.
